# Using Computer Games to Support Mental Health Interventions: Naturalistic Deployment Study

**DOI:** 10.2196/12430

**Published:** 2019-05-09

**Authors:** Hidde van der Meulen, Darragh McCashin, Gary O'Reilly, David Coyle

**Affiliations:** 1 University College Dublin School of Computer Science Dublin Ireland; 2 University College Dublin School of Psychology Dublin Ireland

**Keywords:** mental health, eHealth, mHealth, adolescents, children, computer game, treatment, cognitive behavioral therapy

## Abstract

**Background:**

Recent research has highlighted *naturalistic uptake* as a key barrier to maximizing the impact of mental health technologies. Although there is increasing evidence regarding the efficacy of digital interventions for mental health, as demonstrated through randomized controlled trials, there is also evidence that technologies do not succeed as expected when deployed in real-world settings.

**Objective:**

This paper describes the naturalistic deployment of Pesky gNATs, a computer game designed to support cognitive behavioral therapy (CBT) for children experiencing anxiety or low mood. The objective of this deployment study was to identify how therapists use Pesky gNATs in real-world settings and to discover positive and negative factors. On the basis of this, we aimed to derive generalizable recommendations for the development of mental health technologies that can have greater impact in real-world settings.

**Methods:**

Pesky gNATs has been made available through a not-for-profit organization. After 18 months of use, we collected usage and user experience data from therapists who used the game. Data were collected through an online survey and semistructured interviews addressing the expectations and experiences of both therapists and young people. Thematic analysis was used to identify key themes in the interview and survey data.

**Results:**

A total of 21 therapists, who used Pesky gNATs with 95 young people, completed the online survey. Furthermore, 5 therapists participated in the follow-up interview. Confirming previous assessments, data suggest that the game can be helpful in delivering therapy and that young people generally liked the approach. Therapists shared diverse opinions regarding the young people for whom they deemed the game appropriate. The following 3 themes were identified: (1) stages of use, (2) impact on the delivery of therapy, and (3) customization. We discuss therapists’ reflections on the game with regard to their work practices and consider the question of customization, including the delicate balance of adaptable interaction versus the need for fidelity to a therapeutic model.

**Conclusions:**

This study provides further evidence that therapeutic games can support the delivery of CBT for young people in real-world settings. It also shows that deployment studies can provide a valuable means of understanding how technologies integrate with the overall mental health ecosystem and become a part of therapists' toolbox. Variability in use should be expected in real-world settings. Effective training, support for therapist autonomy, careful consideration of different approaches to customization, the reporting of deployment data, and support for communities of practice can play an important role in supporting variable, but effective, use.

## Introduction

### Youth Mental Health

Recent years have witnessed significant progress in technologies designed to support improved mental health in adults [1-5]. Although the evidence on the potential of technology to support interventions with young people is growing [6,7], the body of research in this area is more limited. This stands in sharp contrast to the stated objectives of the international health policies, where the prevention and treatment of mental health problems is a central objective [8,9], and where young people are identified as particularly vulnerable [10] and requiring specific attention [11,12]. Evidence suggests that 50% of mental disorders emerge by 14 years of age and 75% present before 24 years of age [13]. The Global Burden of Disease figures reveal that young people aged between 10 and 24 years represent 27% of the world’s population within which poor mental health is the leading cause of disability [14]. Unaddressed mental health difficulties in early life are associated with at least a threefold increase in the odds of having a mental health disorder in later life [15]. Early intervention is widely encouraged to save lives, prevent young people from falling into crisis, and help in reducing the need for expensive long-term interventions in adulthood [13]. Patel et al argue that “more research is urgently needed to improve the range of affordable and feasible interventions, as most mental-health needs in young people are unmet, even in high-income countries” [10].

### Therapeutic Games

This paper focuses on Pesky gNATs, a computer game designed to support the delivery of cognitive behavioral therapy (CBT) for young people experiencing low mood or anxiety [16]. Pesky gNATs is located among a growing body of research on the use of serious games in mental health [17-19]. Other examples include SPARX [20], Rainbow SPARX [21], The Feel Good Island [22], Op Volle Kracht [23], Personal Investigator [24], Ricky the Spider [25], Treasure Hunt [26], and The Journey [27]. Many of these games have been the subject of small-scale evaluations. Pesky gNATs itself builds on a previous game, gNATs Island (see evaluation in [28]). However, larger-scale studies have also been conducted. A randomized controlled noninferiority trial of SPARX demonstrated that it was a viable alternative to usual care for adolescents presenting with depressive symptoms in primary care. By reducing the costs and clinical resources associated with treatment, it has the potential to help in addressing unmet demand [29]. A randomized controlled trial (RCT) of The Feel Good Island demonstrated the potential of a serious game to support CBT for adults with intellectual disabilities [22].

### Naturalistic Evaluations of Digital Interventions

Pesky gNATs is currently being evaluated through a multisite RCT in primary care psychology services in Ireland [30]. That study will allow us to understand the controlled use of Pesky gNATs and provide robust evidence on the efficacy of the game. This paper explores a different approach, focusing on the use of the game in real-world settings, that is, outside of controlled settings. We believe this approach is complementary to traditional RCTs and can help maximize the impact of digital interventions.

The importance of alternative evaluation strategies for digital interventions, including *naturalistic* studies, is increasingly recognized by researchers [18,31,32]. In part, this reflects a misalignment between the timescales of RCTs and the rapid advancement and subsequent obsolescence of technologies. It also reflects the limitations of traditional RCTs in understanding the impact of digital interventions when deployed in real-world contexts. Discussing the evolving, interdisciplinary nature of research on digital interventions, Blandford et al argue that “If interactive digital health interventions are to be sustainable, and a good investment...then it is essential to invest time and effort in ensuring that they are incorporated within broader care delivery systems in ways that work well for professionals, patients, and others involved in care” [33]. Increasingly, researchers argue that implementation data should be routinely analyzed to assess technologies and their fit in the real-world [32,34].

These arguments are part of the ongoing discussions within the literature on how to optimize the impact of digital technologies within day-to-day services, with Fleming et al suggesting that mental health technologies are not reaching or engaging the intended users post release [34]. A comparison of real-world usage and that found in controlled studies of MoodGYM, found that adherence of users in the RCT [35] was significantly higher than adherence in public users [36]. This is particularly important given the evident correlation between adherence and efficacy [37]. Another large-scale RCT assessing MoodGYM and Beating the Blues also found unexpected results. In contrast with previous RCTs, no significant differences were found between the digital interventions and usual care [38].

Similar discrepancies can be found within serious games literature. Poppelaars et al [39] described a school-based trial with adolescents reporting subclinical depressive symptoms in which they compared the games Op Volle Kracht, SPARX, and a combination of the 2 with a wait-list group. The study showed no significant differences of depressive symptoms among the 4 groups [39]. The authors found comparable results in other studies about Op Volle Kracht but found conflicting results with a similar study by Wijnhoven et al, which reported positive outcomes for the program [40]. Poppelaars et al suggested that the differences may be attributable to stricter study inclusion criteria used by Wijnhoven et al. In a similar comparison regarding SPARX, contrasting results were found between the studies by Poppelaars et al [39] and Fleming et al [20]. It is argued that these differences can be caused by the different participant environments (school-going vs school-excluded). These findings demonstrated that small differences in study criteria can have a significant impact on outcomes.

As noted above, this paper is grounded in the naturalistic deployment of Pesky gNATs. The aim of this deployment was to identify how therapists used Pesky gNATs in real-world settings. Building on this, the broader aim of this paper was to contribute toward generalizable recommendations for the design of mental health technologies that can have greater impact in real-world settings.

### Pesky gNATs

Pesky gNATs is a 3-dimensional (3D) computer game that helps to teach young people CBT concepts and skills [16,41]. Here we briefly outlined the key aspects of the game and its theoretical basis. The CBT content of Pesky gNATs is further described in Multimedia Appendix 1, with a more detailed description available in the study by O’Reilly [41].

**Figure 1:**
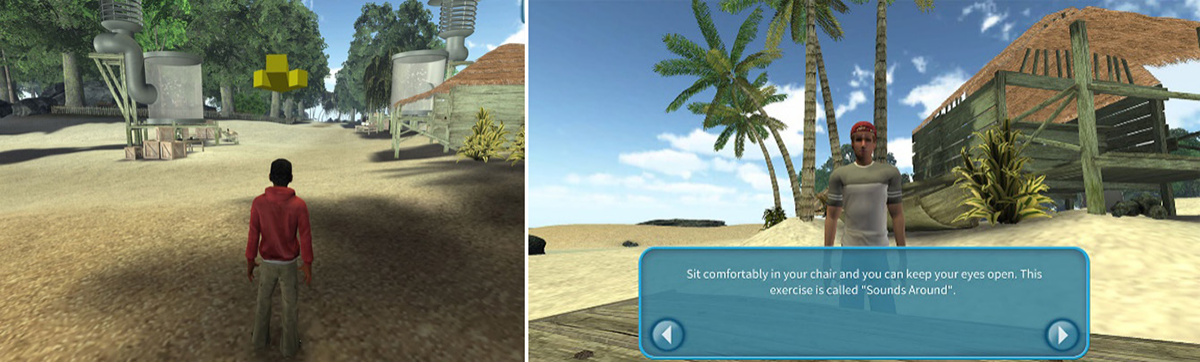
The player controls a character, exploring an island (left), and meets characters who explain cognitive behavioral therapy using comprehensible metaphors. The player also meets Ben, the Beach Dude (right), who teaches mindfulness and relaxation skills.

Pesky gNATs is played by young people with clinically significant anxiety or low mood, during face-to-face therapy sessions with a suitably qualified mental health professional. In sessions, the young person and therapist sit together at a computer, but the young person has full control of the mouse and keyboard. They navigate through a 3D world where they meet a series of characters who introduce CBT concepts using spoken conversation, embedded animations, videos, and questions regarding the young person’s own situation (Figure 1). The in-session computer game is supplemented by a mobile phone app that aims to support young people in transferring what they learn in therapy to their home, school, and day-to-day life [16].

In addition to the game and app, the overall program includes 7 hours of online training videos for therapists. This provides a description of the concepts in the program and a full walk-through of the game and app, illustrating how they are typically used during therapy. Our intention with Pesky gNATs was to assist mental health professionals in delivering a genuinely *cognitive* CBT intervention, filtered through the ideas of developmental psychology and learning theory [42-46], packaged in a computer game that is played within the supportive context of a therapeutic relationship.

The gNATs of the game’s title is a play on the CBT concept of negative automatic thoughts. Each level of the game introduces young people to a single CBT concept, which is explained and illustrated by a game character. The child, with assistance from the therapist, applies the CBT concept to the anxiety or low mood difficulties they are experiencing. The game characters set the young person a challenge of putting that concept into practice in his or her daily life. The young person then has the opportunity to learn 1 of 9 behavioral activation, relaxation, or mindfulness skills chosen by the therapist to best meet his or her current needs. Each game level takes about 50 min to play, which is the typical length of standard child therapy appointments. Completion of between session tasks is supported through the Pesky gNATs app or a hard copy workbook. Overall, Pesky gNATs is intended to provide a coherent and consistently delivered child-friendly introduction to CBT through its programmed content, combined with an individually nuanced, personal delivery supported by the judgement and accompanying conversations young people have with their therapists.

## Methods

### Overview

This study focused on therapists’ experiences of using Pesky gNATs during the first 18 months of deployment. The software and online training materials had been made available on a not-for-profit basis on a website [16]. Before accessing the software, therapists were required to complete an online registration process that included a screening questionnaire. The questionnaire was manually screened by GOR to determine if applicants had appropriate qualifications in the delivery of CBT for young people. Therapists meeting the requirements received a username and password that enabled them to access a secured website, where they could purchase a license for Pesky gNATs, download the software, and access the training materials.

### Data Collection and Analysis

Data were collected through an online survey and optional semistructured interviews with therapists. The survey was implemented using LimeSurvey. All submissions were anonymous unless participants opted in for the follow-up interview, in which case contact details were required. Qualitative and quantitative data were collected with the aim of capturing how Pesky gNATs was used, identifying positive and negative factors, and exploring the expectations and experiences of therapists and young people. The survey was divided across the following topics: (1) use of Pesky gNATs, (2) helpfulness of Pesky gNATs, (3) therapy style, (4) pacing of Pesky gNATs, (5) game design, (6) customization, and (7) future developments. The inclusion of specific questions on customization reflected arguments that modularization or customization might be beneficial in supporting naturalistic uptake of digital interventions [47].

Follow-up semistructured interviews were conducted via phone or video call and followed the remote interview procedures described by Braun and Clarke [48]. Interviews addressed similar topics to those in the online survey but allowed us to explore participants’ attitudes and experiences in greater detail. HM conducted and audio recorded the interviews, which lasted 40 to 60 min. Verbatim transcriptions of the recordings were made. Each transcript was manually checked for accuracy.

As the topics of the open questions in the survey had a strong overlap with those discussed in the semistructured interviews, we analyzed and presented the qualitative results from both sources together. Thematic analysis was used to identify codes and themes across the entire dataset, following the guidelines described by Braun and Clarke [48]. Furthermore, 10% of the coded data were screened by a different researcher (DMC), and disagreements were discussed until consensus was reached.

Ethical approval for this study was granted by the Research Ethics Committee at University College Dublin (reference LS-E-17-150-VanderMeulen-Coyle). Survey participants gave consent before the first question, and written consent was obtained before the start of the interviews.

### Participants

An invitation to participate in the online survey was emailed to all Pesky gNATs license holders. In total, 91 survey invitations were sent. A total of 21 participants completed the survey with 5 opting in for follow-up interviews. On average, survey participants had a Pesky gNATs license for 429 days (SD 257 days) at the point of data collection (June 2018). In total, they had started the program with 95 young people (mean young participants per therapist=4.5 [SD 4.2]), ranging from 1 to 16 children per therapist. They estimated that they had completed the full 7 session program with 39 children (mean=1.9 [SD 2.6]), ranging from 0 to 12 programs completed per therapist.

## Results

### Overall Opinions

On the basis of their experiences in delivering therapy with Pesky gNATs, therapists were asked to estimate the age range for which they deemed the program most appropriate. Responses varied from 6 to 13 years as the minimum age (mean=8.8 [SD 1.5] years) and 10 to 21 years as the maximum age (mean=14.0 [SD 2.5] years). In addition, estimations of the play time for individual Pesky gNATs sessions ranged from 15 to 60 min (mean=36 [SD 11]).

**Figure 2:**
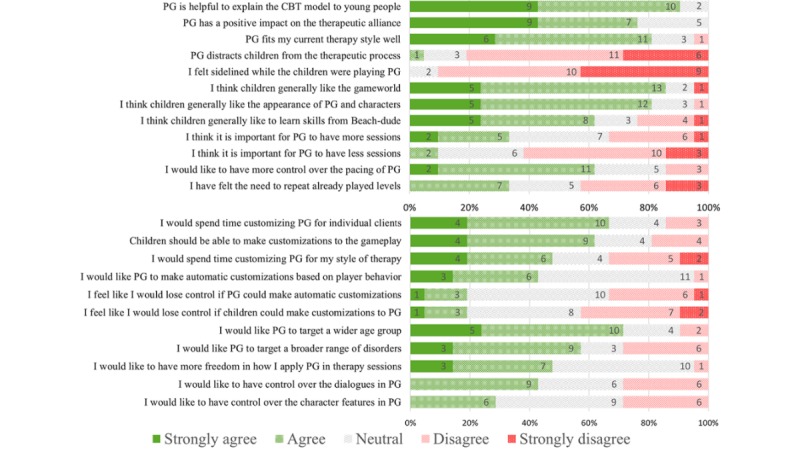
Therapists’ (n=21) responses to Likert questions about Pesky gNATs, its fit to their therapeutic practice, estimation of the children’s experiences, and attitudes related to the pacing, customization, and target group. Numbers represent absolute number of occurrences. CBT: cognitive behavioral therapy; PG: Pesky gNATs.

Likert questions captured the therapists’ overall opinions on the helpfulness of the game, children’s experience, pacing of the game, and attitudes regarding different approaches to customization. The aggregated responses are shown in Figure 2.

Overall, the therapists rated the helpfulness of Pesky gNATs positively, suggesting that it helped to explain the CBT model to young people and indicating that it fitted well with their therapy style. They indicated that the program had a positive impact on the therapeutic alliance. It was generally considered not to distract from the therapeutic process, and the therapists did not feel sidelined when children controlled the game. Therapists felt that the children generally liked the game world and characters and responded well to exercises introduced by game characters.

Overall, the therapists expressed positive attitudes regarding the potential for customizations, indicating that they would spend time making customizations to support individual clients. They also indicated a preference to see the game extended to target a wider age group and were generally positive about the idea of the game targeting a broader range of disorders. Although more positive than negative responses were given, overall attitudes were less clear with regard to customizations to their own therapy style, options to modify the overall pacing of the game, repeat or change the number of game sessions, or modify characters. Therapists were also more neutral regarding the potential for young people themselves or the computer to make automatic customization.

### Thematic Analysis

**Figure 3:**
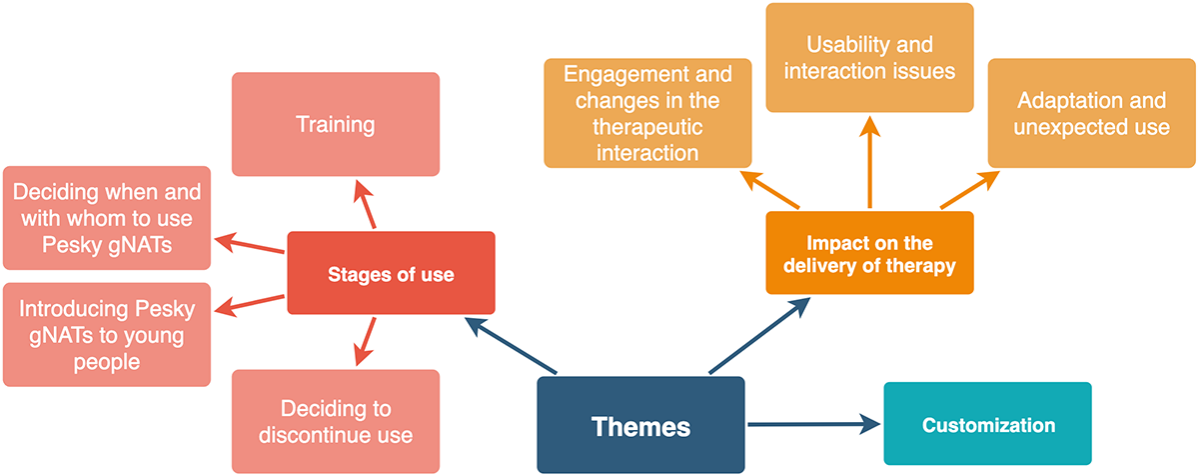
Overview of the identified themes and subthemes.

Thematic analysis of survey data and interviews identified the following 3 key themes: stages of use, impact on the delivery of therapy, and customization. As illustrated in Figure 3, 2 main themes contained multiple subthemes. For example, the stages of use theme included subthemes on training, deciding when and with whom to use Pesky gNATs, the approaches taken when introducing the game, and equally important, decisions regarding when to stop using the game.

#### Theme 1: Stages of Use

##### Training

The majority of therapists indicated that they completed the full online training. The training was valued and considered helpful in understanding the concepts and approaches to delivering therapy with Pesky gNATs. However, therapists also noted that the practical experience in using the game was important in gaining full value from the program:

My experience has been that becoming familiar with the material in advance and using the support available on the website means that I am in a position to anticipate and/or respond to any signs that a young person needs more time to explore ideas and their own feelings during a session.

P20, survey

A minority of therapists reported completing most or some of the training, of which one said that the game is easy to work with without the training. Several noted time constraints, with one explaining that they had insufficient time to complete training regarding the mobile phone app and therefore decided to focus on material relating to face-to-face sessions and supplement this with the paper workbook.

##### Deciding When and With Whom to Use Pesky gNATs

As per the recommended useage guidelines of Pesky gNATs, it was typically used to address depression and anxiety. Age, willingness to engage in face-to-face therapy, the complexity of the difficulties faced, and the intellectual abilities of the young people were key considerations in decisions to use the game. However, there were significant variations across therapists regarding the young people and situations where the game was deemed most appropriate. Some felt it was best suited for mild to moderate anxiety, but less fitting for severe cases. For example, a therapist explained that young people with severe psychological difficulties needed something simple as they might not have the patience or ability to engage with the game content. They continued that the game had been very effective in addressing low mood of children with regular ability levels. In contrast, another therapist described complex cases where Pesky gNATs was used after confirming the children’s abilities and understanding of concepts needed for the game:

Some people on the autism spectrum and children with attachment disorder and borderline learning disabilities as well. So very complex children...I would do a preassessment of their actual understanding of thoughts, feelings, and behaviors...The other thing would be, have they got the intellectual ability to cope with the game and the patience to manage the game.

P12, interview

Importantly, therapists reflected that the decisions to use Pesky gNATs were not taken in isolation of other options. They described it as one of the tools they used to support the delivery of interventions. Although some therapists appeared to consider Pesky gNATs as a default option, others explained that the game was only introduced as an alternative when young people were reluctant to engage with the therapy. Overall, the therapists’ decisions regarding the use of the game appeared to evolve with experience, with some describing decision-making steps:

...the first thing I would consider is, are they going to jell well with the material? Do they see the purpose of that material and what it’s trying to say...[Secondly] any learning difficulties. The third consideration...have I got the time as an educational psychologist because our time is so limited?

P4, interview

One of the most surprising findings was that several therapists felt Pesky gNATs was not suitable for young people who regularly played computer games, as they might have different expectations from a game and miss the excitement of more action-oriented gameplay, described by one therapist as *killing things*.

##### Introducing Pesky gNATs to Young People

The video training emphasized that the manner in which Pesky gNATs is introduced is important. In particular, although we aimed to produce a high-quality game, Pesky gNATs cannot, nor is it intended to, compete with commercial games in terms of state-of-the-art graphics and the scale of the gameplay. The training suggested that therapists introduce the game to young people in a way that managed expectations and explained that Pesky gNATs was likely be a different experience to regular games. Participants confirmed the importance of this approach. One explained that they introduced the game by contrasting it to regular talk-based therapy. Another explained how an appropriate introduction was effectively used with a 17-year old, who might otherwise have felt too old for Pesky gNATs:

I used it with one pupil who was 17 years old because we almost looked at it with a bit of humor, almost tongue-in-cheek and alright, this is going to be quite corny and I was making a point. Giving them that little caveat, I think it was still helpful for her.

P4, interview

Therapists typically had traditional sessions with children before introducing Pesky gNATs. Some had 1 or 2 intake sessions before using the game, whereas others indicated it could take up to 3 or 4 sessions. One therapist described how it was useful to have a contract as part of the introduction and that this could help to maintain engagement:

I have made a couple of contracts with boys that I think could use it. Like, you do the first four sessions and then we’ll talk about it and if you absolutely do not like it, we’ll do something else and everybody’s chosen to finish it. Some people have tolerated it, I should say, but they did get some things out of it. Using that contract with them really works.

P7, interview

Another therapist explained that it had been useful to introduce Pesky gNATs to parents by having them join the first session. This allowed parents to learn what happens in therapy and develop a better understanding of how to support their children.

##### Deciding to Discontinue Use

Pesky gNATs was not always helpful. When children were dissatisfied or when the therapists noticed that the game was not helping, it was discontinued. In some cases, children did not understand the CBT concepts, because of either comprehension issues or distraction. A therapist described a boy who switched to *gamer mode* and wanted to get to the fun and challenging parts of the game quickly. He had lost focus on the therapeutic concepts and had to be slowed down to focus, which had become a frustration in itself:

It was like he’d switched to gaming mode instead of CBT mode. So, for him it didn't help the therapeutic relationship in that session because it got in the way.

P10, interview

In contrast, some young people found the game boring or childish. For example, a therapist found that 16- and 17-year-old boys might feel *too cool* for the game. It is important to recognize these cases and where appropriate, discontinue its use.

Interestingly, the decision to stop using the game could be helpful to the overall therapeutic process. A therapist described a case where she introduced the game to a girl who was reluctant to engage in therapy. The girl also disliked the game and they discontinued its use. However, the therapist described how an improved relationship then emerged:

Even though we didn’t use the game, I think it actually helped move the therapeutic relationship on, because we were able to have a conversation about the game.

P10, interview

Often it was a collaborative decision to stop using Pesky gNATs and try alternatives. A therapist described conversations with young people about why the game did not work and encouraged them to try it for at least two sessions. If the game remained unhelpful, the paper workbook with the same concepts was used instead, effectively leaving out the technology but keeping the narrative.

#### Theme 2: Impact on the Delivery of Therapy

##### Engagement and Changes in the Therapeutic Interaction

Most participants considered the game helpful and valued it as part of their practice. Therapists were somewhat surprised by how much young people learned and felt it was a *really helpful way* to introduce concepts that were ordinarily difficult to explain. Others described how the game and narrative helped with engagement, describing how navigating and exploring the island together was a good way to break the ice, build rapport, and maintain engagement:

They were excited about coming back to play Pesky gNATs, which people have not found that kind of excitement for them. The parents have not ever seen them excited to come back.

P7, interview

Changes in the traditional face-to-face interaction were identified as an important factor in improving engagement. Therapists described how the intensity that some young people experienced in face-to-face conversations was reduced:

I really like the idea of Pesky gNATs because you’ve got this tool to mediate that interaction and to build on. And I guess, to use as a prompt and an extension so the child’s hearing the messages from a different place, as well as from you.

P10, interview

All three boys would have been reluctant to engage with paper-based activities without the motivation and context provided by the game. In each case, the program was helpful to lessen the intensity of one-to-one sessions. One young person particularly enjoyed some of the “goofy” aspects, such as the celebratory dance and the humor (intended or not) was very helpful to him.

P35, survey

As illustrated by this quote from P35, the adoption of a digital medium and elements of humor were also seen as important. However, although this attitude was common, it needs to be contrasted with other cases where some older children and gamers were alienated by the game-based approach and felt the humor was not *cool*.

Contrasting reactions were also evident in more specific functionality within Pesky gNATs. The use of standardized questionnaires provided a powerful example. Most therapists and children were happy with the in-game use of psychological scales to identify difficulties and visualize progress. However, in a minority of situations they were seen as detrimental. One therapist described how displaying questionnaires’ results had the potential to put undesired labels on children:

I find it hard to then have a conversation around, “We’re not looking at you being OCD, we’re not thinking about a diagnosis. What we’re thinking about is you having rigid and recurring thoughts and how we can address that”, but it’s hard to address something when you’re given a label.

P10, interview

The mobile phone app was mostly perceived as useful for homework between sessions, but some therapists reported difficulties with children’s engagement. There was too much competition for attention on phones, and some children did not own a phone, did not have access to their parents’ device, or could only install apps with their parent’s permission.

##### Usability and Interaction Issues

Several therapists noted that they encountered logistical difficulties in installing the software or loading and saving files on restricted organizational systems. When using the game, some felt that young people found the graphics and animations disappointing. Sometimes this was because of graphical glitches, such as a slow frame rate on older machines. Updating or upgrading the visual appearance of the game was suggested frequently when therapists were asked about potential improvements.

Age- and disorder-specific interaction difficulties were also noted. A therapist described how a 10-year old with anxiety difficulties could take a long time to complete text-based answers to questions in the game, feeling the answers had to be spelled and punctuated perfectly, which caused further anxiety. They suggested replacing some text input with multiple-choice answers or emoticons to simplify the interactions.

Some therapists suggested that the overall experience could be enhanced by adding more fun to parts of Pesky gNATs *to take a break if it feels too content heavy*. For example, having more mini-games as rewards after talking to characters and learning new concepts. In addition, therapists recommended additional gamification of therapeutic exercises:

More game type engagement could be very valuable. Games to identify and match gNATs to thoughts could be good. Further gamifying the therapeutic challenges could be very valuable and motivating.

P22, survey

The majority of therapists felt the structure of sessions and pacing of CBT concepts were appropriate. However, some did encounter difficulties with younger children’s comprehension of concepts. Therapists noted that they would like the flexibility to go back and repeat previous levels. This is not currently possible; however, one therapist explained that using the paper workbook to revisit materials provided a workable alternative. Pacing was also considered from an interaction perspective. In addition to finding game characters, one therapist explained that children liked swimming and playing with beachballs and kicking them in the water and asked if there could be more similar interactions. However, as with other aspects of the game, there were variations in experiences. A minority felt that navigation on the island was too slow for some children and a quicker way to navigate would be an improvement.

##### Adaptation and Unexpected Use

In some cases, therapists supplemented the content of the game. One described how the homework exercises in Pesky gNATs did not match the immediate challenges of the child, and alternative homework was created that remained close to the game narrative:

She would get anxiety that there was always a burglar breaking into the house and so then, she didn’t want to sleep in her own bed. So, we did homework around some classic exposure therapy and helping her parents. So, it didn’t dovetail exactly with the game. Although, we did call it her Pesky gNat. We named it. It was this burglar gNat.

P5, interview

When working with young children it was not uncommon for therapists to involve parents or guardians. Although Pesky gNATs was not designed to explicitly support such involvement, several therapists did involve parents. For example, parents were invited to join the first session or were updated via email. A therapist explained that the homework exercises were useful to engage the family of a child. They felt that the program could be extended to more explicitly engage parents and educate them about the therapy sessions, so their support can be optimized. In contrast, others were more cautious, noting that some parents showed low engagement in the therapy of their children or that parents were part of the therapeutic difficulties. One explained that care should be taken when designing technology that requires children to engage their parents, as it might set up the child to fail if parents are resistant to this engagement.

#### Theme 3: Customization

Typically, therapists were open and positive to potentially customizing Pesky gNATs. Therapists would consider adjusting the game for individual children, as long as it was time-efficient, for example, repeating levels, adjusting in-game examples of thoughts and feelings, or difficulty of challenges and dialogues. Nevertheless, one therapist explained that they would not know how to make the game more engaging as they were not game developers. Another therapist worried that too much freedom could raise issues with the fidelity of the CBT process:

I don’t think I would...because I know me well enough to know that I would skip over parts that are necessary, maybe to rush it a little bit and the one thing I can do now is say, “we’ve got to go from beginning to end. It’s set up that way, I can’t just do the fun things with you”. That is a safeguard for me and the kids.

P7, interview

Some therapists liked the user-centered approach and favored children’s ownership in therapy. They felt that allowing children to make changes to the gameplay and avatars could support engagement. Other therapists warned of potential distractions of such approaches because of the psychological difficulties that can arise:

I would just be concerned that children using it, particularly if they suffer with anxiety, need support, and to feel continued. Perhaps making changes together, if the child is a bit older?

P8, survey

Automatic customization in game mechanics by the computer were also considered. For example, mechanics that adjusted the difficulty of challenges based on the progress and performance of players. Some therapists were skeptical of automatic adjustments, fearing that it might impede professional judgements. Some also felt they had insufficient technical experience to appraise automatic adjustments.

## Discussion

### Principal Findings

Although our ongoing RCT [30] will provide more robust results on the clinical efficacy of Pesky gNATs, the naturalistic results reported here provide further evidence that serious games can support mental health interventions in real-world settings. The results indicate that the helpfulness of Pesky gNATs can be explained by factors including child-friendly, metaphor-driven narratives; playful elements; the fact that it is played alongside a therapist; and the changed therapeutic dynamics the game supports. Notably, we found evidence that serious games may be less appropriate for those who regularly play games (*gamers*).

Viewed more broadly, this deployment study also allowed us to identify generalizable recommendations relevant to the wider community, including researchers and designers seeking to maximize the impact of digital technologies in real-world settings. These include the following:

- Expect variability in use and support therapist autonomy
- New technologies become part of an overall toolbox
- Report implementation data, but also support a community of practice
- Carefully consider different approaches to customization and adaptability
- Deciding when and how to discontinue use is also important and can be helpful

#### Expect Variability in Use and Support Therapist Autonomy

Variability in use is perhaps the clearest message to emerge from our study. The young people for whom the therapists felt Pesky gNATs was most appropriate varied widely in age and psychological difficulties. There was also significant variability with regard to when therapists chose to introduce the game (eg, as a default or only with reluctant young people).

Variability is likely to reflect the reality of technology use in the real-world and as such it should be anticipated and where possible supported. Recognizing the role of the therapist as a decision-maker and considering how this autonomy can be supported is an important design consideration. Pesky gNATs was specifically intended to support therapists’ existing practices and recognize the value of therapeutic relationships. The game does not seek to replace therapists; rather it is aimed at subtly changing the dynamics of face-to-face interventions and introducing CBT concepts in a child-friendly manner, while ensuring that the therapist retains control and guides the overall intervention.

It is important to note that in this context, variable use does not indicate suboptimal use. Equally, we do not advocate inconsistent or inappropriate use. For example, access to Pesky gNATs is subject to manual screening of therapists’ experience and qualifications. Although this process is time-consuming and subject to abuse, we believe it is essential. Furthermore, although therapists in our study did not agree on the situations in which Pesky gNATs was most appropriate, they described careful individual decision-making processes regarding its use. The ability to understand the CBT concepts in the game and regular, reliable meeting schedules played an important role in such decisions. The online training for Pesky gNATs also provided guidance on such decisions. More broadly, researchers should accept the ongoing responsibility to gather data that helps to establish the boundaries of acceptable use and update and evolve guidelines accordingly.

#### New Technologies Become Part of an Overall Toolbox

In discussing common misconceptions about mental health technologies, Mohr et al argue that “research literature typically describes and evaluates mental health technologies as if they were products” [32]. Rather than viewing new technologies as products that can mediate change in and of themselves, they argue it is more appropriate to view them as technology-enabled services, which serve to support the overall service or therapeutic process. Supporting the autonomy of therapists will be an important factor in advancing this new perspective. However, it is also important to recognize that individual programs or services will not exist in isolation. Rather, they will become part of the broader toolbox from which therapists will choose.

This deployment study clearly showed that Pesky gNATs was not used in isolation. Rather, therapists considered it as one option among many. For some it was a preferred option, whereas others only used it when young people were resistant to more traditional face-to-face approaches. When it was used, therapists often used it in combination with other approaches. Sometimes these alternatives directly mirrored activities in Pesky gNATs. For example, some young people preferred to do mindfulness exercises directly with their therapist, instead of using the game. On other occasions, therapists supplemented Pesky gNATs with activities entirely separate to the program, based on the emerging needs of young people (eg, guided exposure). Providing a paper workbook as an alternative or backup to the game and mobile phone app was also an important part of the therapist toolbox.

#### Report Implementation Data, but Also Support a Community of Practice

As noted in the Introduction, there is growing recognition that implementation data should be routinely reported [32,34]. We support such recommendations and believe that shared deployment data have clear benefits for the research community. In this context, it is critically important that both the successes and failings of systems are shared. It is also important to report how technologies fit with the broader ecosystem, which is multifaceted and involves overlapping technical, organizational, and social factors. This is illustrated by the difficulties some therapists experienced when installing Pesky gNATs on organizational computers. In addition, some young people did not have their own smartphones or were dependent on parental permission to install the app. Sharing such experiences will enable us to develop generalizable theories and design principles that support more impactful technologies, for example, guidelines for customization or supporting therapist autonomy.

Beyond the research community, we also recommend a further use of deployment data—supporting the creation of communities of practice. Therapists in our study clearly valued the online training. However, they explained that the experience of using the game was critical. Although each therapist needs to gain this experience first-hand, there is a strong argument for supporting online communities where experiences of using new technologies can be shared. Specific examples with regard to Pesky gNATs include techniques for introducing the game and managing expectations, or the use of therapeutic contracts. The creation of communities of practice may also be effective in encouraging early adopters of digital technologies to become active, participatory co-designers in working with developers and researchers to improve interventions.

#### Carefully Consider Different Approaches to Customization and Adaptability

Our study explored 3 distinct approaches to customization. Across all approaches, therapists clearly recognized the need for systems to maintain fidelity of a core therapeutic model. The following are the customization approaches:

- *Customization by therapists* was generally viewed positively, although an important distinction can be drawn between changes to therapeutic content versus changes to interaction-specific features. For example, the option of adjusting the in-game examples of thoughts and feelings or content of in-game dialogues was viewed favorably. Therapists were less willing or confident in their ability to modify game-based or technical interaction features.
- *Customization by young people* was viewed less positively, even by therapists who favored young peoples’ ownership of therapy. However, specific customizations, such as avatar personalization, were viewed positively. Indeed, recent evidence has suggested that avatar personalization can improve overall engagement [49]; thus, there may be significant value in enabling this form of customization.
- Intelligent *customization by the computer* was viewed favorably by some, but drew skepticism from many therapists, partially because of concerns over a loss in control of the process and also a lack of clear understanding of what intelligent customization would entail. If intelligent customization is to become a feature in mental health technologies, clarity around the targeted adaptation and factors such as transparent and explainable Artificial Intelligence will be critical.

#### Deciding When and How to Discontinue Use Is Also Important and Can Be Helpful

Adherence to study protocols is a defining characteristic of RCTs. Although many RCTs seek to understand and follow up with dropouts, completion of the full intervention, including postintervention data collection, is essential to establish efficacy. In real-world contexts, and when individual technologies are considered as part of a toolbox, the decision to stop using technologies can be viewed more flexibly and with less focus on adherence. If a technology alienates a young person to the extent that they withdraw from therapy, this would be a significant cause for concern. However, evidence from this study suggested that therapists and young people often made joint decisions to stop using the game. In such cases, discussions about why the young person disliked the game—and what alternative approaches might be useful—were often helpful in and of themselves.

This more flexible view of technology—where systems are engaged for as long as they are useful and ongoing use is based on users’ emerging needs and reactions—aligns with arguments previously made by Doherty et al [50] in the context of online CBT. More recently, Lederman et al [51] have explored concordance as a valuable perspective through which to view the design of technologies. Here, the overall effectiveness of treatment, support for core treatment goals, and collaborative decision-making involving therapist and clients, again take precedence over strict adherence. The evidence in this deployment study supports Lederman’s argument that concordance is a valuable lens through which to understand the effectiveness of new mental health technologies.

### Limitations

We acknowledge the small sample size and participants’ self-selection bias, as only therapists who had decided to use the program were invited to participate. This study was not designed to provide robust evidence regarding efficacy; no standardized data regarding clinical outcomes were collected. Furthermore, therapists used Pesky gNATs with diverse groups and in diverse contexts and this may have influenced the specific perspectives of therapists.

### Conclusions and Future Work

This study has provided further evidence that serious games can support mental health interventions for young people in real-world settings. More broadly, it provided evidence for the potential of naturalistic deployment studies to complement traditional approaches to evaluation, for example, RCTs. Deployment studies provide a valuable means of understanding how new technologies become part of an overall therapeutic toolbox and help in the development of technologies that are more easily integrated to the mental health ecosystem. Variability in use should be expected in real-world settings. Effective training, support for therapist autonomy, careful consideration of different approaches to customization, the reporting of deployment data, and support for shared communities of practice can play an important role in supporting variable, but effective, use. Future research could include larger-scale deployment studies that bridge the gap between controlled studies and studies in naturalistic settings.

## Appendix

Multimedia Appendix 1 The structure of game levels and cognitive behavioral therapy concepts in Pesky gNATs.

## Abbreviations

3D: 3-dimensional
CBT: cognitive behavioral therapy
RCT: randomized controlled trial

## References

[1] Mohr DC, Burns MN, Schueller SM, Clarke G, Klinkman M (2013). Behavioral intervention technologies: evidence review and recommendations for future research in mental health. Gen Hosp Psychiatry.

[2] Andrews G, Cuijpers P, Craske MG, McEvoy P, Titov N (2010). Computer therapy for the anxiety and depressive disorders is effective, acceptable and practical health care: a meta-analysis. PLoS One.

[3] Andrews G, Basu A, Cuijpers P, Craske MG, McEvoy P, English CL, Newby JM (2018). Computer therapy for the anxiety and depression disorders is effective, acceptable and practical health care: an updated meta-analysis. J Anxiety Disord.

[4] Richards D, Richardson T (2012). Computer-based psychological treatments for depression: a systematic review and meta-analysis. Clin Psychol Rev.

[5] Burns MN, Begale M, Duffecy J, Gergle D, Karr CJ, Giangrande E, Mohr DC (2011). Harnessing context sensing to develop a mobile intervention for depression. J Med Internet Res.

[6] Pennant ME, Loucas CE, Whittington C, Creswell C, Fonagy P, Fuggle P, Kelvin R, Naqvi S, Stockton S, Kendall T, Expert AG (2015). Computerised therapies for anxiety and depression in children and young people: a systematic review and meta-analysis. Behav Res Ther.

[7] Ebert DD, Zarski A, Christensen H, Stikkelbroek Y, Cuijpers P, Berking M, Riper H (2015). Internet and computer-based cognitive behavioral therapy for anxiety and depression in youth: a meta-analysis of randomized controlled outcome trials. PLoS One.

[8] (2011). Great Britain Department of Health and Social Care.

[9] (2013). World Health Organization.

[10] Patel V, Flisher AJ, Hetrick S, McGorry P (2007). Mental health of young people: a global public-health challenge. Lancet.

[11] Buckley S, Cannon M, Chambers D, Coughlan H, Duffy M, Gavin B, Keeley H, McGorry P, Power P, Shiers D (2011). International Association of Youth Mental Health.

[12] (2015). Mental Health Partnerships.

[13] Kessler RC, Berglund P, Demler O, Jin R, Merikangas KR, Walters EE (2005). Lifetime prevalence and age-of-onset distributions of DSM-IV disorders in the National Comorbidity Survey Replication. Arch Gen Psychiatry.

[14] Gore FM, Bloem PJN, Patton GC, Ferguson J, Joseph V, Coffey C, Sawyer SM, Mathers CD (2011). Global burden of disease in young people aged 10-24 years: a systematic analysis. Lancet.

[15] Copeland WE, Adair CE, Smetanin P, Stiff D, Briante C, Colman I, Fergusson D, Horwood J, Poulton R, Costello EJ, Angold A (2013). Diagnostic transitions from childhood to adolescence to early adulthood. J Child Psychol Psychiatry.

[16] O'Reilly G, Coyle D (2015). Pesky gNATs.

[17] Khazaal Y, Favrod J, Sort A, Borgeat F, Bouchard S (2018). Editorial: computers and games for mental health and well-being. Front Psychiatry.

[18] Shah A, Kraemer KR, Won CR, Black S, Hasenbein W (2018). Developing digital intervention games for mental disorders: a review. Games Health J.

[19] Fleming TM, Bavin L, Stasiak K, Hermansson-Webb E, Merry SN, Cheek C, Lucassen M, Lau HM, Pollmuller B, Hetrick S (2016). Serious games and gamification for mental health: current status and promising directions. Front Psychiatry.

[20] Fleming T, Dixon R, Frampton C, Merry S (2012). A pragmatic randomized controlled trial of computerized CBT (SPARX) for symptoms of depression among adolescents excluded from mainstream education. Behav Cogn Psychother.

[21] Lucassen M, Merry S, Hatcher S, Frampton C (2015). Rainbow SPARX: A novel approach to addressing depression in sexual minority youth. Cogn Behav Pract.

[22] Cooney P, Jackman C, Coyle D, O'Reilly G (2017). Computerised cognitive-behavioural therapy for adults with intellectual disability: randomised controlled trial. Br J Psychiatry.

[23] Tak YR, van Zundert RM, Kuijpers RC, van Vlokhoven BS, Rensink HF, Engels RC (2012). A randomized controlled trial testing the effectiveness of a universal school-based depression prevention program 'Op Volle Kracht' in the Netherlands. BMC Public Health.

[24] Coyle D, Matthews M, Sharry J, Nisbet A, Doherty G (2005). Personal Investigator: a therapeutic 3D game for adolecscent psychotherapy. Interactive Tech Smart Ed.

[25] Brezinka V (2013). Ricky And The Spider - a video game to support cognitive behavioural treatment of children with obsessive-compulsive disorder. Clin Neuropsychiatry.

[26] Brezinka V (2008). Treasure Hunt - a serious game to support psychotherapeutic treatment of children. Stud Health Technol Inform.

[27] Stasiak K, Hatcher S, Frampton C, Merry SN (2014). A pilot double blind randomized placebo controlled trial of a prototype computer-based cognitive behavioural therapy program for adolescents with symptoms of depression. Behav Cogn Psychother.

[28] Coyle D, McGlade N, Doherty G, O'Reilly G (2011). Exploratory evaluations of a computer game supporting cognitive behavioural therapy for adolescents.

[29] Rebello TJ, Marques A, Gureje O, Pike KM (2014). Innovative strategies for closing the mental health treatment gap globally. Curr Opin Psychiatry.

[30] O'Reilly G, Coyle D, McCashin D (2019). ISRCTN.

[31] Klasnja P, Consolvo S, Pratt W (2011). How to evaluate technologies for health behavior change in HCI research.

[32] Mohr DC, Weingardt KR, Reddy M, Schueller SM (2017). Three problems with current digital mental health research... and three things we can do about them. Psychiatr Serv.

[33] Blandford A, Gibbs J, Newhouse N, Perski O, Singh A, Murray E (2018). Seven lessons for interdisciplinary research on interactive digital health interventions. Digit Health.

[34] Fleming T, Bavin L, Lucassen M, Stasiak K, Hopkins S, Merry S (2018). Beyond the trial: systematic review of real-world uptake and engagement with digital self-help interventions for depression, low mood, or anxiety. J Med Internet Res.

[35] Christensen H, Griffiths KM, Farrer L (2009). Adherence in internet interventions for anxiety and depression. J Med Internet Res.

[36] Christensen H, Griffiths KM, Korten AE, Brittliffe K, Groves C (2004). A comparison of changes in anxiety and depression symptoms of spontaneous users and trial participants of a cognitive behavior therapy website. J Med Internet Res.

[37] Twomey C, O'Reilly G (2017). Effectiveness of a freely available computerised cognitive behavioural therapy programme (MoodGYM) for depression: meta-analysis. Aust N Z J Psychiatry.

[38] Gilbody S, Littlewood E, Hewitt C, Brierley G, Tharmanathan P, Araya R, Barkham M, Bower P, Cooper C, Gask L, Kessler D, Lester H, Lovell K, Parry G, Richards DA, Andersen P, Brabyn S, Knowles S, Shepherd C, Tallon D, White D (2015). Computerised cognitive behaviour therapy (cCBT) as treatment for depression in primary care (REEACT trial): large scale pragmatic randomised controlled trial. Br Med J.

[39] Poppelaars M, Tak YR, Lichtwarck-Aschoff A, Engels RC, Lobel A, Merry SN, Lucassen MF, Granic I (2016). A randomized controlled trial comparing two cognitive-behavioral programs for adolescent girls with subclinical depression: a school-based program (Op Volle Kracht) and a computerized program (SPARX). Behav Res Ther.

[40] Wijnhoven L, Creemers D, Vermulst A, Scholte R, Engels R (2014). Randomized controlled trial testing the effectiveness of a depression prevention program ('Op Volle Kracht') among adolescent girls with elevated depressive symptoms. J Abnorm Child Psychol.

[41] O'Reilly G (2018). Pesky gNATs! Using computer games and smartphone apps to teach complex cognitive behavioural therapy and mindfulness concepts to children with mental health difficulties. The Use of Technology in Teaching and Learning.

[42] Bandura A, Walters R (1977). Social Learning Theory.

[43] Beck J (2011). Cognitive Behavior Therapy: Basics and Beyond.

[44] Dunn J (1988). The Beginnings of Social Understanding.

[45] Flavell J (1963). The developmental psychology of Jean Piaget. The University Series in Psychology.

[46] Vygotsky L, Cole M (1978). Mind in Society.

[47] Fleming TM, de Beurs D, Khazaal Y, Gaggioli A, Riva G, Botella C, Baños RM, Aschieri F, Bavin LM, Kleiboer A, Merry S, Lau HM, Riper H (2016). Maximizing the impact of e-therapy and serious gaming: time for a paradigm shift. Front Psychiatry.

[48] Braun V, Clarke V (2013). Successful Qualitative Research: A Practical Guide for Beginners.

[49] Birk MV, Mandryk RL (2019). Improving the efficacy of cognitive training for digital mental health interventions through avatar customization: crowdsourced quasi-experimental study. J Med Internet Res.

[50] Doherty G, Coyle D, Sharry J (2012). Engagement with online mental health interventions: an exploratory clinical study of a treatment for depression. Proceedings of the SIGCHI Conference on Human Factors in Computing Systems.

[51] Lederman R, Gleeson J, Wadley G, D’alfonso S, Rice S, Santesteban-Echarri O, Alvarez-Jimenez M (2019). Support for carers of young people with mental illness: design and trial of a technology-mediated therapy. ACM Trans Comput Hum Interact.

