# Supplementary material for: Using Computer Games to Support Mental Health Interventions: Naturalistic Deployment Study
Source: JMIR Ment Health. 2019 May 9;6(5):e12430. doi: 10.2196/12430 (PMC6707559; doi:10.2196/12430)
Supplement: Multimedia Appendix 1 [file mental_v6i5e12430_app1.pdf]

Game Level	Central CBT Concept
**Game Level 1:** Thoughts Feelings & Behaviours	**Session concept:** Thinking-Feeling-Behaviour (TFB) go together.<br>**In session activity:** Young person with assistance from therapist creates a positive and a negative TFB from the last 24 hours.<br>**Between session task:** record one positive and one negative TFB.
**Game Level 2:** Cognitive Monitoring	**Session concept:** Some thoughts are unhelpfully negative and we usually don't notice them.<br>**In session activity:** Negative Automatic Thoughts (NATs) animated as gNATs or little flies and young person reviews 6 different types (species) of gNATs and learns how to record them using a gNAT trap. Young person reviews their negative TFB to see if it contains a gNAT.<br>**Between session task:** Between session task to trap some gNATs focusing on times of anxiety or low mood.
**Game Level 3:** Cognitive Monitoring	**Session concept:** Young person is introduced to 5 more common species of gNAT.<br>**In session activity:** They review their gNAT trapping to-date. Young person responds to their gNATs with some PATs (positive automatic thoughts).<br>**Between session task:** to trap some more gNATs focusing on times of anxiety or low mood.
**Game Level 4:** Cognitive Restructuring	**Session concept:** Introduction of cognitive restructuring presented as gNAT swatting.<br>**In session activity:** Young person applies 4 gNAT swatting questions to (i) consider evidence for and against their thoughts, (ii) to consider alternative ways of looking at things, (iii) to brain-storm alternative plans for similar situations and (iv) to pick and test a new plan.<br>**Between session task:** to trap and swat gNATs focusing on times of anxiety or low mood.
**Game Level 5:** Negative Core Belief Identification	**Session concept:** Introduction of negative Core Beliefs and review of common examples.<br>**In session activity:** Young person gathers up all of the gNATs they trapped so far and hunts them back to a suitable Hive (Core Belief).<br>**Between session task:** to hunt gNATs back to a Hive.
**Game Level 6:** Negative Core Belief Re-appraisal	**Session concept:** Socratic questioning of identified core belief.<br>**In session activity:** Young person considers evidence for and against their core belief in the areas of self, family, school, friendships and other. Young person decides if Core Belief is true or not (Hive splatting) and builds a positive belief called a Bee Urself Hive.<br>**Between session task:** Continued as a between session task.
**Game Level 7:** Relapse Prevention	**Session concept:** Relapse prevention.<br>**In session activity:** The young person identifies signs of relapse and plans their response. The young person also develops a healthy life plan setting positive goals in the areas of 1. Fun 2. Personal Goals 3. Having a Purpose 4. Emotional and Physical Health 5. Being Me. 6. Having People in My Life.
